# The Early Diagnosis and Early Treatment of Acute Anterior Poliomyelitis*Read before the Mississippi Valley Medical Society, October, 1892.

**Published:** 1892-12

**Authors:** Archibald Church

**Affiliations:** Chicago, Ill.; Professor of Neurology, Chicago Polyclinic; Lecturer on Insanity, etc., Chicago Medical College


### THE EARLY DIAGNOSIS AND EARLY TREATMENT OF ACUTE ANTERIOR POLIOMYELITIS.*

BY ARCHIBALD CHURCH, M. D., CHICAGO, ILL.

Professor of Neurology, Chicago Polyclinic ; Lecturer on Insanity, etc., Chicago Medical College.

Aside from trauma, perhaps the most prolific source of deformity and of physical incapacity from useless limbs is acute inflammation of the anterior spinal horns of gray matter. It is believed that a considerable portion of this deformity and this helplessness may be obviated by an early diagnosis of the spinal trouble, and prompt treatment to prevent the distortions which ultimately and usually follow in its wake. This must be sufficient apology for bringing forward a rather trite subject.

That the diagnosis of paralysis from destruction of the cornual gray matter of the cord is not always made early is demonstrated too frequently in every clinic for nervous troubles where children with flabby, wasted, pendulous extremities are brought by anxious parents, with the story that the physician who first saw the child made light of the trouble, attributing it to worms, or cutting teeth, or some fever, and assuring them that the child would outgrow the disability. Very often the initial fever of the malady is mistaken for some trifling ailment, and when the child shortly afterwards is found paralyzed, the family rather naturally lose confidence in the medical adviser who had given an unguarded prognosis, and whose chagrin is readily imagined.

*Diagnosis.*—The most susceptible period of life for the onset of this disease is during the latter half of the first year, the predisposition rapidly diminishing thereafter, so that over three-fifths of all cases are encountered under four years of age. The inability of these little patients to express their feelings in language adds greatly to the diagnostic problem, and leads often to a misinterpretation of conditions that are mainly subjective. Nearly every systematic writer on this

*Read before the Mississippi Valley Medical Society, October, 1892.

topic lays emphasis on the absence of sensory disturbances in the early stage of the malady as well as later, but notes in older children and in the rare cases occurring in adult life, the presence of rheumatoid pains and cutaneous tenderness, with a tendency, however, to attribute them to an associated neuritis, or to some intercurrent malady; but that sensory disturbance does occur early has been noted too often, even in infants, to be attributed to coincidental difficulties, or to be described as an unusual feature. If this hyperæsthetic condition of the involved limbs be carefully sought, it will be found, in all probability, very rarely absent during the first few days, or even the first fortnight, of the disease, and lends great assistance in deciphering the case.

The onset of the disease presents considerable variation, which again adds to our difficulties. Usually there is a febrile stage with a temperature of from 101 to 102 deg., peevishness, restlessness, loss of appetite and general indication of discomfort, lasting from a few hours to a week or ten days, during which inactivity in certain limbs is noticed, but unless the medical man be on the alert, is attributed to the general illness, which in turn is mistaken for intestinal disturbance, or any of the numerous slight ailments marked by low temperatures that beset childhood. In some instances the onset is extremely abrupt, a complete paralysis of limbs being found without any antecedent *malaise*. Possibly the child has gone to sleep in apparent health and awakes paralyzed. In other instances there is much mental excitement and generalized convulsions with vomiting, which misleads the attention in the direction of cerebral mischief and lends coloring to the supposition that the succeeding palsy is of inter-cranial origin. Cerebral palsy, however, is of rather more sudden development, and the attending convulsions are of a Jacksonian type, involving only a portion of the body, a single limb, or perhaps only one side of the face. They are usually repeated at short intervals for a number of hours or may extend over several days, and are marked by profound depression and often by stupor, while the convulsions in question are complete in their distribution, single or few in number, and not related to cortical irritation and localization.

Now, during the febrile stage, if the child is handled carefully, it will be noticed that the manipulation of certain limbs gives rise to manifestations of distress not provoked by similar interference on the opposite side, and the sensitive limbs will either be found already inactive or will shortly present a palsied state. At times a fall or injury, to which infant life is so liable, will give rise to the impression that the local tenderness is the result of some such accident and must be investigated on surgical lines. Indeed, almost as a rule, when the paralysis is fully developed, some one is accused of carelessness in handling the child, and unjust blame often falls upon the nurse. Lack of mobility from injury is likely to involve a single large joint to which the tenderness is also confined; while in cornual involvement, especially at first, the paretic state is much more extensive. Even when but a slight permanent palsy remains, as for instance, impairment of the tibialis anticus, the initial paralysis usually involves the entire extremity, and the tenderness here insisted upon is also of equally wide distribution.

A peripheral neuritis, except as the result of severe injury, with which of course it would have anatomatical relations, is positively rare in infancy, and a multiple neuritis even rarer. When present, it is of gradual onset, marked by pain, extreme tenderness and slight, or no, febrile movement. The resulting atrophy is of later development and usually is manifest in the skin, nails and other dormal structures, which are spared by acute poliomyelitis. The symmetry of distribution in multiple neuritis at once characterizes it and contrasts it with the monoplegic tendency of cornual inflammation, while the antecedent clinical history of an infectious disease, of metalic poisoning, or of syphilis, or other dyscrasia is highly suggestive though not distinctive of the peripheral disease in which, too, the paralysis is of tardy appearance, requiring weeks to reach its maximum. In poliomyelitis the palsy is most pronounced at the very first, is very rarely hemiplegic in distribution, and much more rarely of a paraplegic type.

At the end of a fortnight the character of the palsy can be absolutely determined by testing the muscles with a faradic current. Any simple battery will answer the purpose, and in the unaffected limbs we have a perfect standard of comparison. All involved muscles will show a lowered activity when stimulated by this form of electrical current, which determines at once the extent of palsy and of cord involvement while giving information of great value as to the outlook. Any muscle that shows the least response may be expected to regain a fair share of its strength, while those totally inert may confidently be expected to remain in some degree paretic and to waste. Knowing, then, the muscles that will be spared, their tendency to contraction can be met, and in this is the principle of treatment upon which too much stress cannot be laid.

Without going into details regarding the muscle groups usually involved, it may be said that the cervical and lumbar enlargements of the cord are the preferred locations for the central lesion, and drop-wrist and drop-foot the most usual result, with pronounced tendency to contractures and flexion at the knee, hip and elbow. The limb is rarely so completely involved as to result in a permanent flail-like appendage.

The essentials, then, for an early diagnosis are a slight fever, local tenderness, flacidity of muscles and a failure to respond to a faradic current strong enough to cause contraction in the sound limbs. The tendon reflexes are abolished also very early, but as they are difficult to obtain in infants, their investigation has not been insisted upon.

*Treatment.*—If one is fortunate enough to make the diagnosis during the fever, that condition will be variously met, though there is reason to think that the real nature of the disease is the result of intoxication by a ptomaine or leucomaine, with an elective action upon the spinal gray matter, and from this point of view salicylates or mercury may be advisable. At this time the child should be kept as much as possible on the side or face, and very mild sinapisms to the spine with brisk catharsis seem indicated, as well as other measures of proved value in myelitis. Usually, however, the fever has subsided and the paralysis has been noted before the case is clearly diagnosed, and it is at this juncture that the greatest help can be afforded in the way of preventing deformities.

The conditions for consideration in local treatment are these: the normal balance of the muscles passing a given articulation is disturbed by the weakening of flexors and extensors; either from gravity or by the action of imposed muscles, or both, the joints are disposed to be placed in, and to maintain unusual positions; joint surfaces and ligaments in infancy quickly yield and the malposition becomes mechanically fixed. A muscle that is allowed for a long time to retain a contracted state tends to become inextensile. A muscle in a stretched position always acts at a disadvantage, because in that condition its anatomical opponent is in the best mechanical position to act vigorously. The important deduction is to maintain normal positions and relations in the affected extremities.

In the somewhat analagous condition of wrist-drop from lead poisoning, it is found that when all other treatment has practically failed, the continued employment of splints to maintain the joints in a straight position, or better, in one of extension, is followed by very marked improvement. The reasons seem obvious: the joint surfaces are thus re-accustomed to their normal relations, and the weakened extensors are given the advantage of position. This advantage is especially required in the palsy poliomyelites, because the implicated muscle rarely regains its full power, and being temporarily deprived of all strength, should find the most favorable conditions for its action as it gradually regains tone. Considering in such a case the ankle-joint after the expiration of several months, we find the calf muscles shortened, the toe pointing downwards and inwards, the anterior tibial group of muscles, the ones usually involved, wasted and presenting but a little of their former strength, and this exerted in almost a straight line, without leverage against the strongly acting and contractured sural mass which has besides every mechanical advantage. The little strength in the anterior muscle has slowly developed, but could it have found the joint in a normal position and been able to respond to its diminished trophic and motor spinal influence, its prospects for development and usefulness would have been greatly improved.

Therefore, to reiterate, the normal position of involved limbs is from the first to be persistently maintained. This, however, is not always an easy task; but by ingenuity, patience and intelligent assistance of the mother, it can be accomplished. The affected limb should be dressed warmly, and this warm dressing may be made the means of the indicated mechanical support. In the lower extremity, a well-fitting, long stocking attached to a waist can be used. Two whalebone or vulcanite dress stays sewed to the front and back of this stocking support the knee, and a very light elastic band to the foot will, by its proper arrangement, correct the tendency to drop-foot and turned ankle. A similar arrangement can be used for the upper extremity, the stocking terminating in a mitten and being secured to a waist at the shoulder. The comparative immobilization of the hip joint is much more difficult, but an upward extension of the dress stays to the armpit answers fairly well. In some cases a light brace may be used with advantage, or ordinary fracture dressings.

The next item of importance is to maintain the nutrition of the paralysed muscles until the trophic influence of the cord is restored, that it may find all conditions favorable for its usually much impaired activity. This is to be accomplished, except in the muscles whose central apparatus has been completely destroyed by the disease, by means of massage, friction and electricity, for which it is their only indication. The rather hazy notions prevalent concerning some mystic relations between electricity and nerve force has led to the empirical use of the battery in all paralytic states, but if it be understood that its only utility is to maintain the activity of muscle tissue and to conserve thereby its responsiveness to nerve control, its use will be made much more precise and its limitations more clearly apprehended. These muscles early lose their excitability to faradic or induced electricity, and consequently the galvanic current to which they respond with abnormal readiness must be used. Six to ten active cells, a broad negative sponge on the sternum or sacrum and a smaller positive sponge on the muscles is an appropriate arrangement. Care must be used not to overwork the muscles, and most of all not to alarm and distress the infant, as thereby more harm than good results. The contractions which only occur on contact of the sponge with the limb should not exceed a dozen at each daily seance, but may be increased later as voluntary power returns, or faradic response reappears, both of which are favorable indications for the individual muscles so reacting. It is a doubtful expedient to intrust electrical treatment to the parents or others, however intelligent.

Frequently repeated massage and frictions are of like importance and for the same purpose. These can be carried out by lay people with great satisfaction. It is highly important in these manipulations to thoroughly stretch all muscles which show, or are likely to show, any tendency to permanent shortening, to passively put all joints through their widest range of motion and to confine the massage principally to the palsied muscles. Frequent sponge bathing is also beneficial and may be combined.

The use of strychnia can only be of value by its tonic action and its physiological effect on the cord, hence its hypodermic employment, which has found advocates, is a needless cruelty, but its careful and persistent administration by the mouth, after the active process has subsided, is often of value. With electricity and massage it should not be used during the first two or three weeks. Any subnormal condition of the general health and any dyscrasia, lack of hygienic surroundings and of proper diet should of course receive early and persistent attention.

